# Determinants of Slow-Wave Activity in Overweight and Obese Adults: Roles of Sex, Obstructive Sleep Apnea and Testosterone Levels

**DOI:** 10.3389/fendo.2018.00377

**Published:** 2018-07-12

**Authors:** Lisa L. Morselli, Karla A. Temple, Rachel Leproult, David A. Ehrmann, Eve Van Cauter, Babak Mokhlesi

**Affiliations:** ^1^Sleep, Metabolism and Health Center, University of Chicago, Chicago, IL, United States; ^2^Division of Endocrinology and Metabolism, Department of Internal Medicine, University of Iowa Carver College of Medicine, Iowa City, IA, United States; ^3^Section of Endocrinology, Department of Medicine, University of Chicago, Chicago, IL, United States; ^4^Section of Pulmonary and Critical Care, Department of Medicine, University of Chicago, Chicago, IL, United States

**Keywords:** obstructive sleep apnea, sex differences, slow wave sleep, slow wave activity, delta activity, testosterone, spectral analysis

## Abstract

**Background:** Slow-wave activity (SWA) in non-rapid eye movement (NREM) sleep, obtained by spectral analysis of the electroencephalogram, is a marker of the depth or intensity of NREM sleep. Higher levels of SWA are associated with lower arousability during NREM sleep and protect against sleep fragmentation. Multiple studies have documented that SWA levels are higher in lean women, compared to age-matched lean men, but whether these differences persist in obese subjects is unclear. Obstructive sleep apnea (OSA), a condition associated with obesity, is more prevalent in men than in women. Sex differences in SWA could therefore be one of the factors predisposing men to OSA. Furthermore, we hypothesized that higher levels of testosterone may be associated with lower levels of SWA.

**Objective:** The aim of the current study was to identify sex differences in the determinants of SWA in young and middle-aged overweight and obese adults.

**Methods:** We enrolled 101 overweight and obese but otherwise healthy participants from the community (44 men, 57 women) in this cross-sectional study. Participants underwent an overnight in-laboratory polysomnogram. The recordings were submitted to sleep staging and spectral analysis. Sex differences and the potential contribution of testosterone levels were evaluated after adjusting for age, body mass index and race/ethnicity.

**Results:** OSA was present in 66% of men and in 44% of women. After adjustment for differences in age, race/ethnicity and BMI, the odds ratio for OSA in men vs. women was 3.17 (95% CI 1.14–9.43, *p* = 0.027). There was a graded inverse relationship between the apnea-hypopnea index (AHI) and SWA in men (β = −0.21, *p* = 0.018) but not in women (β = 0.10, *p* = 0.207). In a multivariate regression model, higher testosterone levels were independently associated with lower SWA in men after controlling for age, race/ethnicity and apnea-hypopnea index (β = −0.56, *p* = 0.025).

**Conclusion:** Increasing severity of OSA was associated with significant decrease in sleep intensity in men but not in women. Higher testosterone levels were associated with lower sleep intensity in men. Men with higher testosterone levels may therefore have lower arousal thresholds and higher ventilatory instability in NREM sleep, and be at greater risk of OSA.

## Introduction

Slow-wave activity (SWA) in non-rapid eye movement (NREM) sleep, also known as delta activity, is a marker of the depth or intensity of NREM sleep. SWA may be quantified by spectral analysis of the electroencephalogram (EEG) in the low frequency range typical of slow waves (0.75–4.5 Hz) (1, 2). SWA measurements are highly reproducible from night to night in a given individual (3–5). Higher levels of SWA are associated with lower arousability during NREM sleep and protect against sleep fragmentation due to external or internal disturbances (6). Multiple studies have documented that SWA levels are higher in women, compared to age-matched men (7–9) but the majority of the findings were obtained in lean adults.

Observational studies have reported significant transient reduction of obstructive sleep apnea (OSA) severity during slow wave sleep (SWS), the deepest stage of NREM sleep, which is associated with higher levels of SWA (10, 11). Rigorous mechanistic studies examining the neuromechanical properties of the upper airway during NREM sleep have shown that the upper airway is indeed less collapsible during SWS (12, 13). A higher arousal threshold during SWS, which is partly determined by the intensity of NREM sleep, i.e., the level of SWA, has also been recognized as one of the factors leading to OSA improvement during SWS (14, 15).

Estimations of OSA prevalence have typically found that the disorder is much more common in men than in women, particularly in young and middle-aged adults (16, 17). Obesity is an important and well-recognized risk factor for OSA. However, women with OSA are typically heavier than men when matched for OSA severity (18–20). Thus, being a woman appears to offer a relative protection against the development of OSA, even in the presence of more severe obesity. The reasons for this sex disparity in OSA risk have not been clearly identified, and may lie in anatomical, neuronal, and/or hormonal differences (21). In particular, whether sex differences in SWA are also present in overweight and obese adults and are associated with differences in OSA presence and severity has not been systematically evaluated.

The potential role of testosterone levels, which are much lower in women than in men, in the regulation of SWA and in the risk of OSA is an important related question. Testosterone levels are known to be modulated by sleep duration, sleep restriction and sleep quality in men (22–24). Several small studies have examined the impact of OSA on testosterone levels in men and some have reported decreased testosterone concentrations in men with OSA compared to controls (25–27). On the other hand, multiple reports of exogenous testosterone administration triggering or worsening OSA have been published (28–32). However, it should be noted that participants received supraphysiologic doses of testosterone in all of these studies except one (32). In the latter study, the worsening of OSA severity was only transient (32). Conversely, pharmacologically induced reduction of testosterone in healthy men has been shown to increase breathing stability during NREM sleep, potentially leading to a reduction in the risk of OSA (33, 34), while a small study in premenopausal women demonstrated a decrease in breathing stability after <2 weeks of transdermal testosterone administration resulting in male testosterone levels (35).

The aim of the present study was therefore to examine sex differences in sleep architecture, assessed by visual scoring of all-night polysomnographic (PSG) laboratory recordings and quantitative EEG analysis, and to identify factors associated with SWA in overweight and obese men and women, with and without OSA. Additionally, we explored the possible contribution of endogenous testosterone levels to individual differences in SWA in both men and women.

## Participants and methods

Subjects aged 20–50 years with body mass index (BMI) >25 kg/m^2^ were recruited from the community between 2008 and 2014 using written advertisements inviting participation in research studies on sleep and metabolism. Sleep complaints or symptoms of OSA were not used as selection criteria for the study. Exclusion criteria were: self-reported habitual sleep duration of <6 h per night or more than 10 h per night, chronic insomnia, any sleep disorder other than OSA, any prior or current treatment for OSA (upper airway surgery, CPAP therapy, oral appliances or supplemental oxygen), history of substance abuse, hypnotic or psychotropic medications, tobacco use, caffeine intake above 300 mg per day, alcohol intake above 10 drinks per week, pregnancy, hormonal therapy, liver disease, renal insufficiency, heart failure, cancer, chronic infectious diseases, neurological or psychiatric diseases, diabetes, shift work within the last 3 months and travel across more than two time zones in the 4 weeks before the sleep recording. All female participants were premenopausal and were not taking hormonal contraceptives.

Study participants had a physical examination, and a complete medical history was obtained. Height and weight were measured. Self-reported race/ethnicity was recorded. One hundred and thirty participants underwent an overnight in-laboratory PSG with a minimum recording time of 7.5 h. Twenty nine subjects were excluded from analysis due to artifacts in the EEG (*n* = 22), total sleep duration <5 h (*n* = 5) or recording missing from server (*n* = 2). EEG recordings devoid of significant artifacts and with total sleep time of at least 5 h were obtained in 101 participants (44 men and 57 women). The following morning, fasting blood samples were taken to measure total and free testosterone and sex-hormone binding globulin.

### Assays

Plasma total testosterone was measured by a direct RIA kit (Coat-A-Count; Siemens Medical Solutions USA, Inc; Malvern, PA, USA) that has been validated against a liquid chromatography/tandem mass spectrometry method (36) with functional sensitivity of 10 ng/dl and normal range of 312–1240 ng/dl in men and 19–70 ng/dl in women. Total testosterone values were not obtained in 2 men and 1 woman. The plasma free testosterone concentration was computed as the product of total testosterone and percentage free testosterone, which was determined directly in plasma by competitive protein-binding assay with a sensitivity of 3 pg/ml (37, 38). Free testosterone levels were not obtained in 7 men and 1 woman. Sex hormone binding globulin (SHBG) was measured by an in-house assay based on a competitive protein binding procedure (37, 38). Intra-assay variation coefficients for testosterone, free testosterone and SHBG were 6, 6, and 19%, respectively, in men, and 12, 13, and 14%, respectively, in women.

### Sleep analysis

Sleep recordings were performed in the laboratory using a digital EEG acquisition system (Neurofax EEg-1100 A, Nihon Kohden, Tokyo, Japan). Lights-off time was tailored to match self-reported habitual bedtime (± 1 h) and total recording time was at least 6 h. Surface electrodes were used to collect the EEG signals and the acquisition montage included, in addition to a vertex central referential lead and one ground lead, two central EEG leads (C_3_ and C_4_), two occipital leads (O_1_ and O_2_), two mastoids leads (A_1_ and A_2_), one vertical and one horizontal electro-oculogram (EOG), one bipolar submental chin electromyogram (EMG), and one bipolar electrocardiogram (ECG). The presence or absence of OSA was evaluated by measuring oronasal airflow signal by thermal flow sensor and nasal pressure transducer, respiratory effort by thoracic and abdominal piezoelectric belts or resistance inductive plethysmography and arterial oxygen saturation by finger pulse oximetry. Limb movements were also recorded from one bipolar tibial EMG to exclude individuals with movement disorders. Polysomnographic recordings were visually scored in 30-s epochs by an experienced professional sleep technologist considering stages W, N1, N2, N3, and R in adherence with standardized criteria (39). Respiratory events, periodic limb movements and microarousals were scored according to established criteria (39, 40). The apnea-hypopnea index (AHI) was calculated as the total number of obstructive apneas and hypopneas per hour of sleep. Apneas were defined as total cessation of airflow for at least 10 s if respiratory effort was present. Hypopneas were defined as a decrease in nasal pressure signal of ≥30% of baseline, which was associated with either a ≥3% desaturation or an arousal. The presence of OSA was defined by an AHI ≥5 events/h. The microarousal index was defined as the number of microarousals per hour of sleep. The oxygen desaturation index (ODI) was defined as the number of oxygen desaturations ≥3% per hour of sleep.

During acquisition, all EEG signals were filtered between 0.3 and 35 Hz and digitally sampled and stored at 200 Hz with a 16-bit quantization range. Power spectral analysis of the EEG was performed on two central leads (C_3_, C_4_) re-referenced to the linked mastoids references (A_1_ and A_2_). All analyses were performed directly on the original recording files without data format conversion using the PRANA Software Suite (http://www.phitools.com, Strasbourg, France). After removal of muscular, ocular and movement artifacts by automatic and visual inspection, a fast Fourier transform using a 50% overlap between consecutive 4-s elementary windows was computed following the application of a Hanning taper and resulted in a spectral resolution of 0.25 Hz. The spectra of all elementary windows overlapping with artifacts were discarded prior averaging of the elementary spectra on a 30-s epoch basis in order to match the sleep/wake stage scores. When more than 50% of the elementary spectra windows were contaminated, averaging was skipped and the corresponding time series intervals replaced by missing vales in order to preserve continuity in the time series. Absolute power in the delta, theta, alpha and sigma frequency bands (0.75–4.5 Hz, 4.5–8.5 Hz, 8.5–12 Hz, and 12.5–15 Hz, respectively) were calculated by summing up powers of all frequency bins within each band, the lower bin being included and the upper one excluded. Finally, an average of spectral power in each frequency band was calculated using only the 30-epochs from NREM sleep during the first 6 h of sleep. Slow-wave activity in NREM sleep is equivalent to delta power in NREM sleep.

For illustrative purposes, the durations of NREM-REM cycles were normalized to account for individual differences, with each individual NREM period subdivided into 50 equal time bins and each REM period into 20 time bins, as previously described (41). NREM-REM cycles were defined according to the criteria of Feinberg and Floyd (42) and visually verified. For subjects with REM latency >120 min, the termination of the first cycle was identified as the first nadir of SWA. Three subjects had 3 NREM-REM cycles during the night of PSG, while all others had four cycles or more.

### Statistical analysis

Statistical analysis was performed using JMP version 8.0.2 (S.A.S Institute Inc., Cary, NC, USA) and confirmed with SPSS Statistics v20. All group data are expressed as means ± SEM for normally distributed variables, or median (interquartile range) for non-normally distributed variables. Transformation of raw data to achieve normal distribution was performed whenever appropriate prior to statistical analysis. In particular, BMI, AHI and EEG spectral powers were transformed in all analyses using the natural log (Ln). Sex differences for demographic and hormonal characteristics were assessed by Student's *t*-test for continuous variables and chi-square test for categorical variables. Comparisons were considered statistically significant at p < 0.05. Multivariate linear regression models adjusting for age, BMI, and race/ethnicity were fitted to examine sex differences in sleep stages, characteristics of OSA and spectral power in the different EEG frequency bands as well as the interaction sex *x* OSA and sex *x* AHI. When interactions were statistically significant, analyses were repeated separately in men and women. To identify the possible contribution of plasma levels of testosterone and SHBG to the observed sex differences, multivariate linear regression models including the hormonal variable in addition to age, BMI, AHI and race/ethnicity, were fitted separately in men and women.

## Results

### Demographic characteristics and OSA prevalence

In the present cohort of 101 participants, women (*n* = 57) were significantly more obese than men (*n* = 44) (BMI 38.0 ± 1.1 vs. 34.4 ± 0.9 kg/m^2^, *p* = 0.021) and more often African-American (74 vs. 50%, *p* = 0.022). The demographic characteristics of male and female participants, without and with OSA, are summarized in Table 1. Irrespective of sex, participants with OSA were significantly older than those without OSA (37 ± 1 vs. 31 ± 1 years, *p* < 0.0001). Consistent with previous reports (18–20), women with OSA had a higher BMI than men with OSA (median [IQR]: 38.7 [33.1–44.9] vs. 34.3 [32.1–36.8] kg/m^2^, *p* = 0.007). Given these differences in demographics, subsequent analyses were performed adjusting for age, BMI and race/ethnicity. After adjustment, the odds ratio for OSA in men as compared to women was 3.17 (95% CI 1.14–9.43, *p* = 0.027).

**Table 1:** Demographics and hormonal values of the study population.

	**Women no OSA**	**Women OSA**	***p-*value**	**Men no OSA**	**Men OSA**	***p* value**
n	32	25		15	29	
Age (years)	31 ± 1	35 ± 1	**0.006**	30 ± 2	38 ± 1	**<0.0001**
BMI (kg/m^2^)	35.6 (29.9–41.2)	38.7 (33.1–44.9)	**0.042**	31.9 (27.7–38.3)	34.3 (32.1–36.8)	0.501
Race/Ethnicity (%) Non Hispanic White African American Hispanic Asian	22 75 3 0	16 72 12 0	0.391	40 53 7 0	41 48 7 3	0.828
Total testosterone (ng/dl)	33 (25–50)	33 (22–49)	0.708	394 (318–463)	422 (358–551)	*0.095*
Free testosterone (pg/ml)	8 (6–11)	9 (5–12)	0.847	139 (117–169)	139 (116–166)	0.636
SHBG (nM)	26 (17–34)	22 (12–26)	0.160	9 (7–13)	13 (10–16)	*0.070*

*Data are given as mean ± SEM for normally distributed continuous variables, and median (interquartile range) for non-normally distributed continuous variables. For non-normally distributed variables, the results of the statistical analysis reported were obtained after appropriate transformation to normality. To convert total testosterone to nmol/l, multiply by 0.0347; to convert free testosterone to pmol/l, multiply by 3.47*.

*BMI, body mass index; OSA, obstructive sleep apnea. Bold values indicate statistically significant p-values. Italic values indicate trends for statistical significance of p-value*.

Men with OSA showed a trend for higher total testosterone (422[358-551]vs. 394[318-63] ng/dl, *p* = 0.095) and SHBG (13[10-16] vs. 9[7-13]nM, *p* = 0.070) concentrations compared to men without OSA, but free testosterone levels did not differ between these two groups. No difference in total testosterone, free testosterone or SHBG levels were found between women with or without OSA.

### Macro-architecture of sleep

Polysomnographic variables are presented in Table 2. Median (IQR) recording time in the 101 participants was 8 h 06 min (8 h 01–8 h 30). After adjustment, men with OSA had less N3 slow wave sleep compared to men without OSA (11[2-37] vs. 33[7-82]min, *p* = 0.069), although the difference did not reach statistical significance. Moreover, men with OSA had significantly less N3 slow wave sleep than women with OSA (11[2-37] vs. 50[25-65] min, *p* < 0.001). In contrast, in women, N3 slow wave sleep duration was not influenced by the presence of OSA (61[28-82] min in women without OSA vs. 50[24-85] min in women with OSA, *p* = 0.822). Finally, compared to women without OSA, women with OSA had less REM sleep (90 ± 6 vs. 106 ± 5 min, *p* = 0.039).

**Table 2:** Polysomnographic variables.

	**Women no OSA**	**Women OSA**	***p-*****value**		**Men no OSA**	**Men OSA**	***p-*****value**		**Sex × LnAHI interaction *p* value**
			**Unadj**.	Adj.^*^			**Unadj**.	Adj.^*^	
Sleep period time (SPT, min)	465 (453–477)	468 (447–477)	0.587	0.749	490 (453–502)	471 (437–498)	0.721	0.994	0.979
Total sleep time (TST, min)	445 (427–460)	433 (399–455)	*0.053*	0.403	454 (410–491)	433 (404–470)	0.200	0.677	0.638
Sleep efficiency (%)	92 (87–97)	86 (84–93)	**0.041**	0.159	94 (86–96)	88 (83–94)	0.123	0.968	0.764
Sleep latency (min)	15 (11–22)	17 (10–26)	0.692	0.550	14 (7–22)	14 (10–25)	0.445	0.193	0.165
N1 (min)	24 (20–33)	28 (17–38)	0.997	0.707	31 (11–33)	40 (22–51)	**0.012**	0.108	**0.043**
N2 (min)	257 ± 7	258 ± 8	0.939	0.269	278 ± 9	269 ± 8	0.505	0.244	0.813
N3 (min)	61 (28–82)	50 (24–65)	0.563	0.822	33 (7–82)	11 (2–37)	**0.040**	*0.069*	*0.092*
REM (min)	106 ± 5	90 ± 6	**0.037**	**0.039**	106 ± 9	101 ± 6	0.619	0.811	0.424
Wake after sleep onset (min)	18 (9–34)	34 (19–59)	**0.023**	0.252	11 (6–32)	26 (11–53)	0.103	0.548	0.383

*Data are given as mean ± SEM for normally distributed continuous variables, and median (interquartile range) for non-normally distributed continuous variables. For non-normally distributed variables, the results of the statistical analysis reported were obtained after appropriate transformation to normality*.

* *Adjusted for age, LnBMI and race/ethnicity*.

Sleep period time: interval between sleep onset and final morning awakening. Total sleep time: sleep period time minus duration of wake after sleep onset. Sleep latency: time from lights off to sleep onset. Sleep efficiency: total sleep time as percentage of the time allocated to sleep (i.e., the interval between lights off and lights on); N1, NREM stage 1; N2, NREM stage 2; N3, slow-wave sleep; REM, rapid eye movement sleep. Bold values indicate statistically significant p-values. Italic values indicate trends for statistical significance of p-value

### Sex differences in characteristics of OSA

**Figure 1:**
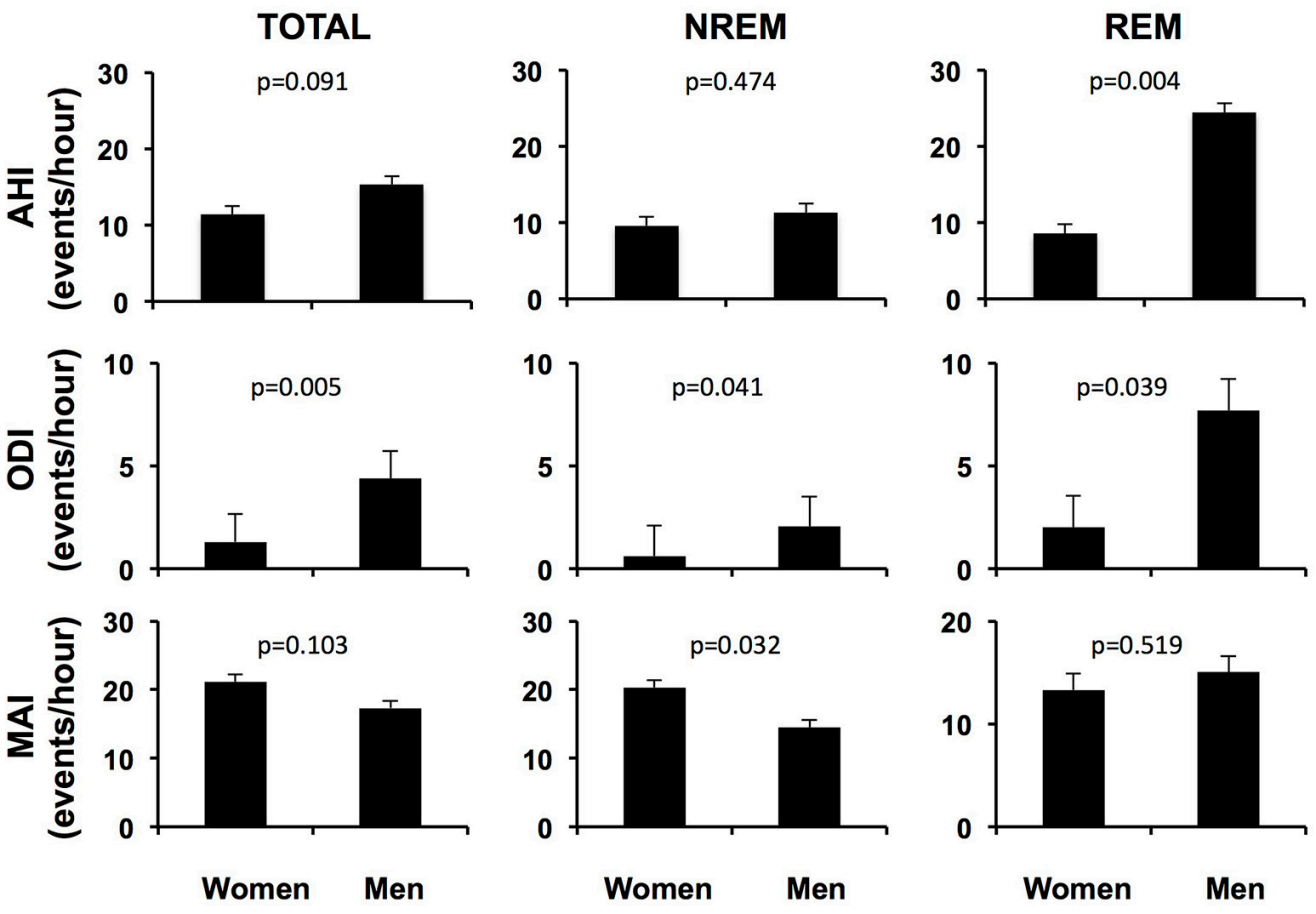
Adjusted respiratory variables and microarousal index of men and women with OSA over the entire night, in NREM sleep and in REM sleep. *AHI* apnea-hypopnea index; *ODI* oxygen desaturation index; *MAI* microarousal index; *NREM* non rapid eye movement sleep; *REM* rapid eye movement sleep. Adjusted for age, BMI and race/ethnicity.

Figure 1 illustrates sex differences in the characteristics of OSA for the entire cohort, after adjusting for age, BMI and race/ethnicity. Men and women had similar total and NREM AHI. However, men had a significantly greater AHI in REM sleep compared to women (24.4 ± 1.3 vs. 8.5 ± 1.3 events/hour, *p* = 0.004), as well as higher total ODI (4.4 ± 1.3 vs. 1.3 ± 1.3 events/hour, *p* = 0.005), NREM ODI (2.1 ± 1.5 vs. 0.6 ± 1.5 events/hour, *p* = 0.041), and REM ODI (7.7 ± 1.5 vs. 2.0 ± 1.6 events/hour, *p* = 0.039). The percentage of sleep time spent below 90% oxygen saturation (T90%) was also higher in men than women (1.04 ± 2.34 vs. 0.07 ± 1.08%, *p* = 0.005), in both REM and NREM sleep. In contrast to variables quantifying hypoxemia, the overall microarousal index was similar in men and women. Although the microarousal index during NREM sleep was higher in women, it was still within the normal range.

### Micro-architecture of sleep

**Figure 2:**
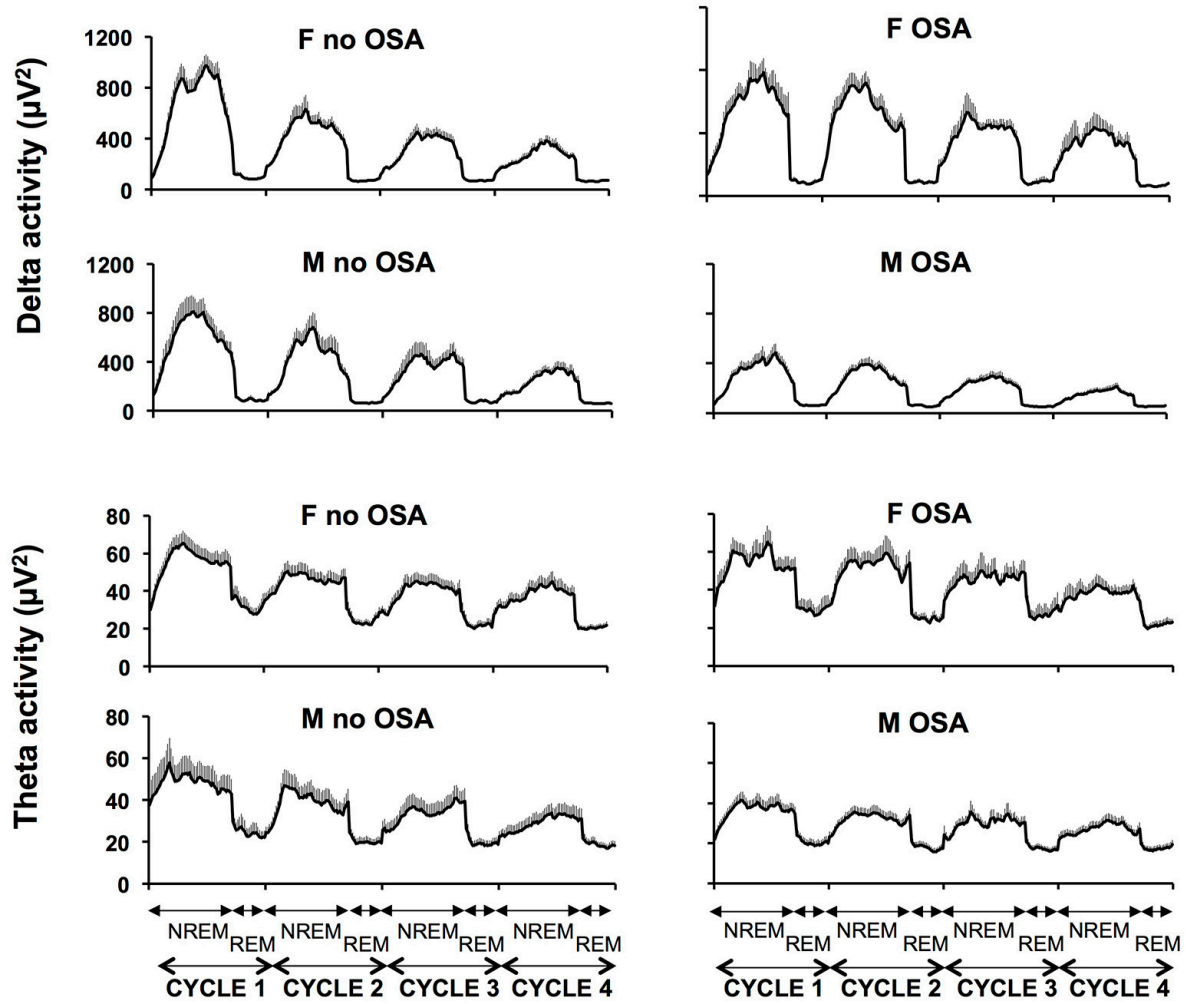
Unadjusted mean profiles (+SEM) of absolute SWA (4 upper panels) and theta activity (4 lower panels) during the first four NREM-REM cycles in women without and with OSA (upper panels) and men without and with OSA (lower panels). *SWA* slow wave activity; *NREM* non rapid eye movement sleep; *REM* rapid eye movement sleep.

Figure 2 illustrates the unadjusted profiles of SWA and theta activity normalized by NREM-REM cycles, in men and women with and without OSA. Profiles of alpha and sigma activity were similar in men and women, with and without OSA (not shown). In unadjusted analyses, the presence of OSA was associated with a 34% lower NREM SWA in men (non OSA 441 ± 51, *vs*. OSA 289 ± 24 μV^2^; *p* = 0.007) but did not significantly impact NREM SWA in women (non OSA 479 ± 37, *vs*. OSA 521 ± 53 μV^2^; *p* = 0.582). As summarized in Table 3, multivariate linear regression confirmed a sex-specific impact of OSA on NREM SWA as reflected by a significant sex *x* LnAHI interaction (*p* = 0.001). For NREM theta activity, a spectral power in an intermediate frequency range that is often contaminated by SWA, a trend for a greater impact of OSA in men than in women (*p* = 0.066 for sex *x* LnAHI interaction) was also present. In contrast, OSA had no significant influence on NREM alpha and sigma activity in either men or women, two power bands generated by neuronal systems distinct from those responsible for SWA.

**Table 3:** Impact of demographic characteristics and OSA on EEG spectral activity in NREM sleep (first 6 h of sleep).

	**SWA (**μ**V**^**2**^**)**		**Theta activity (**μ**V**^**2**^**)**		**Alpha activity (**μ**V**^**2**^**)**		**Sigma activity (**μ**V**^**2**^**)**	
R	0.654		0.483		0.322		0.344	
r^2^	0.428		0.233		0.112		0.118	
r^2^ adjusted	0.392		0.184		0.055		0.062	
*p*-value	<**0.0001**		**0.0003**		*0.075*		*0.060*	
	β **(95% CI)**	***p*****–value**	β **(95% CI)**	***p*****-value**	β **(95% CI)**	***p*****-value**	β **(95% CI)**	***p*****-value**
Sex	0.191 (0.103, 0.280)	**<0.0001**	0.198 (0.087, 0.309)	**0.0006**	0.116 (−0.013, 0.0.245)	*0.078*	0.010 (−0.026, 0.224)	0.119
Age	−0.017 (−0.031, −0.002)	**0.024**	0.004 (−0.014, 0.022)	0.666	−0.001 (−0.021, 0.021)	0.981	−0.012 (−0.032, 0.008)	0.260
LnBMI	0.188 (−0.275, 0.652)	0.422	0.122 (−0.462, 0.706)	0.679	0.049 (−0.711, 0.757)	0.888	0.014 (−0.643, 0.671)	0.966
Race/Ethnicity	−0.237 (−0.324, −0.149)	**<0.0001**	−0.208 (−0.318, −0.098)	**0.0003**	−0.166 (−0.294, −0.038)	**0.012**	−0.207 (−0.330, −0.083)	**0.001**
LnAHI	−0.057 (−0.126, 0.012)	0.105	−0.008 (−0.096, 0.079)	0.851	0.018 (−0.084, 0.119)	0.203	0.012 (−0.086, 0.110)	0.816
Sex *x* LnAHI		**0.001**		*0.066*		0.119		0.798

*β coefficients and p-values from multiple regression models with spectral parameters as dependent variable and sex, age, LnBMI, race/ethnicity, LnAHI and sex x LnAHI interaction as covariates. The results of the statistical analysis reported were obtained after log-transformation of spectral frequency bands*.

NREM, non-rapid eye movement sleep; SWA, slow-wave activity; BMI, body mass index; AHI, apnea-hypopnea index. Bold values indicate statistically significant p-values. Italic values indicate trends for statistical significance of p-value

**Figure 3:**
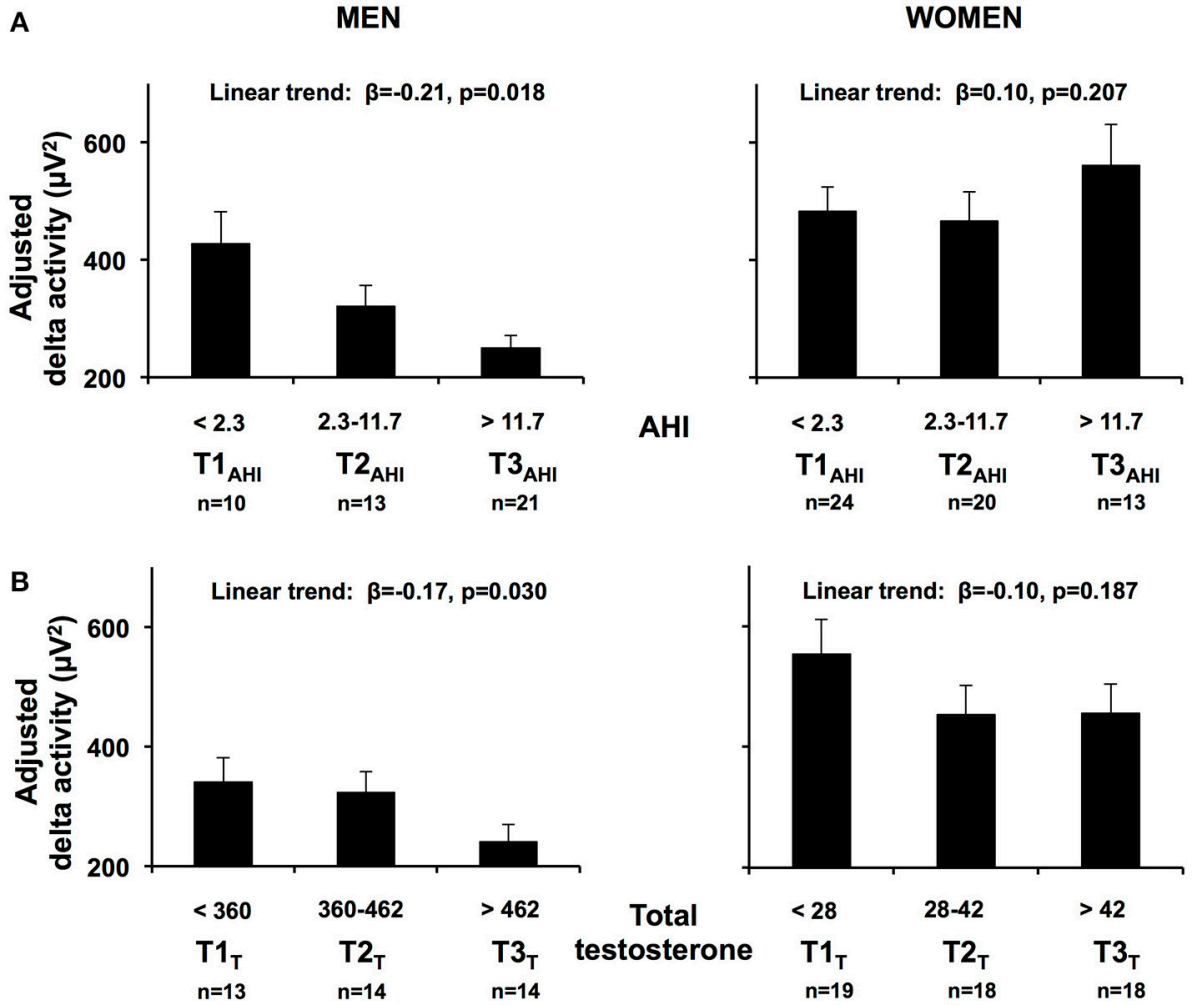
**(A)** Adjusted mean SWA (+SEM) in NREM sleep, in the first 6 h of sleep according to AHI tertiles (T1_AHI_, T2_AHI_, T3_AHI_) in men and women. **(B)** Adjusted mean SWA (+SEM) in NREM sleep, in the first 6 h of sleep according to total testosterone tertiles (T1_T_, T2 _T_, T3 _T_) in men and women. Results obtained from linear regression models including AHI tertiles derived from the total of 101 subjects. Age and BMI were centered at their means. An inverse log transformation was applied to beta coefficients for each AHI tertile to convert from Ln[NREM SWA] to the standard values adjusted for age, BMI and race/ethnicity. Standard errors were obtained by inverse log transformation of the upper and lower 95% confidence intervals of the model estimates and by dividing the difference by four, for each tertile. *SWA*, slow wave activity; *AHI*, apnea hypopnea index.

To facilitate the interpretation of the impact of OSA severity on NREM SWA, we also fitted a linear regression model replacing LnAHI with AHI tertiles derived from the total of 101 subjects (first tertile or T1_AHI_ < 2.3; T2_AHI_ 2.3–11.7; T3_AHI_ > 11.7 events/hour) after adjusting for age, race/ethnicity and BMI. A significant negative linear trend for adjusted SWA was clearly present in men (Figure 3; β = −0.206, *p* = 0.018) such that men in the highest tertile of AHI had SWA levels less than half of those found in men in the lower tertile of AHI. In contrast, in women there was no significant association between AHI tertiles and adjusted SWA (β = 0.104, *p* = 0.207). Similar results were obtained when subdividing the participants according to clinical cut offs of OSA severity (no [AHI < 5], mild [AHI 5–15] and moderate-severe OSA [AHI > 15], data not shown).

### Determinants of NREM SWA in men and women

Because of the significant sex *x* LnAHI interaction present for SWA, we examined the determinants of NREM SWA separately in men and women, and explored the potential roles of circulating total and free testosterone levels as well as SHBG. The upper part of Table 4 describes the results of four multivariate linear regression models predicting NREM SWA in men. Model 1 includes demographic characteristics only, and reveals that age (*p* = 0.004) and race/ethnicity (*p* = 0.001), but not BMI, are significantly associated with SWA levels in this cohort of 20–50 years old overweight and obese men. Introducing AHI (model 2) improved the percentage of variance (r^2^) accounted for by the model; the association with age was no longer significant, while AHI (*p* = 0.002) was independently associated with SWA. The substitution of total testosterone levels for AHI (model 3) increased the r^2^ further, and testosterone levels significantly and negatively correlated with SWA (*p* = 0.005). Since we found no association with BMI in models 1–3, BMI was dropped for the last model to maintain statistical power and we examined simultaneously the contributions of AHI and testosterone levels. SWA remained strongly negatively associated with testosterone (*p* = 0.025), while the strength of the association with age and AHI was considerably reduced. The left lower part of Figure 3 illustrates the association between increasing tertiles of total testosterone levels and SWA in men, after adjusting for age, BMI and race/ethnicity (model 3).

**Table 4:** Determinants of NREM slow wave activity (first 6 h of sleep) in men (top panel) and women (bottom panel).

**MEN**								
**Variables**	**Model 1**		**Model 2**		**Model 3**		**Model 4**	
r	0.579		0.695		0.708		0.728	
r^2^	0.336		0.483		0.501		0.530	
r^2^ adjusted	0.284		0.428		0.445		0.477	
*p*-value	**0.001**		<**0.0001**		<**0.0001**		<**0.0001**	
	**β** **(95% CI)**	***p*** **value**	**β** **(95% CI)**	***p*** **value**	**β** **(95% CI)**	***p*** **value**	**β** **(95% CI)**	***p*** **value**
Age	−0.029 (−0.048, −0.010)	**0.004**	−0.008 (−0.030, 0.013)	0.439	−0.029 (−0.047, −0.012)	**0.002**	−0.017 (−0.036, 0.002)	0.077
Race/Ethnicity	−0.250 (−0.386, −0.115)	**0.001**	−0.249 (−0.370, −0.128)	**0.0002**	−0.257 (−0.377, −0.137)	**0.0001**	−0.258 (−0.371, −0.145)	<**0.0001**
LnBMI	−0.300 (−1.198, 0.597)	0.503	0.233 (−0.635, 1.012)	0.590	−0.433 (−1.306, 0.440)	0.322		
LnAHI			−0.184 (−0.298, −0.071)	**0.002**			−0.106 (−0.224, 0.012)	0.077
LnT					−0.750 (−1.255, −0.244)	**0.005**	−0.560 (−1.043, −0.076)	**0.025**
**WOMEN**								
**Variables**	**Model 1**		**Model 2**		**Model 3**		**Model 4**	
R	0.462		0.475		0.503		0.489	
r^2^	0.213		0.226		0.253		0.239	
r^2^ adjusted	0.167		0.165		0.194		0.178	
*p*-value	**0.006**		**0.010**		**0.005**		**0.008**	
	**β** **(95% CI)**	***p*** **value**	**β** **(95% CI)**	***p*** **value**	**β** **(95% CI)**	***p*** **value**	**β** **(95% CI)**	***p*** **value**
Age	−0.0184 (−0.038, 0.001)	0.059	−0.022 (−0.043, −0.001)	**0.037**	−0.026 (−0.046, −0.006)	**0.014**	−0.029 (−0.051, −0.008)	**0.008**
Race/Ethnicity	−0.220 (−0.351, −0.090)	**0.001**	−0.222 (−0.353, −0.091)	**0.001**	−0.219 (−0.350, −0.088)	**0.002**	−0.29 (−0.339, −0.078)	**0.002**
LnBMI	0.358 (−0.206, 0.922)	0.208	0.242 (−0.376, 0.860)	0.435	0.427 (−0.129, 0.983)	0.130		
LnAHI			0.040 (−0.045, 0.125)	0.353			0.046 (−0.032, 0.124)	0.241
LnT					−0.253 (−0.534, 0.028)	0.077	−0.228 (−0.510, 0.055)	0.112

*Results obtained after log transformation of NREM slow wave activity. NREM non-rapid eye movement sleep; BMI body mass index; AHI, apnea-hypopnea index; T, total testosterone. Bold values indicate statistically significant p-values*.

Similar associations with testosterone were found when free, rather than total concentrations were used. After controlling for age, race/ethnicity, and AHI (model 4), free testosterone level was significantly associated with SWA in men (*p* = 0.012, data not shown). There were no significant associations with SHBG levels in any of the models.

The lower part of Table 4 describes the results of the same four multivariate linear regression models predicting SWA in women. An association of NREM SWA with age and race/ethnicity was observed in all models, while no association between SWA and AHI was present. Testosterone levels were not a significant predictor but the beta coefficient was negative, similar to findings in men, and the significance level suggests the possibility that a trend may be found in a larger sample. The relationship between SWA and increasing tertiles of total testosterone is illustrated for the female participants in the left lower panels of Figure 3.

Of note, while model 4 accounts for nearly 50% of the inter-individual variability in SWA among men, the same covariates account for <20% of the variability among women. In this cohort of overweight and obese but otherwise healthy men and women ages 20–50 years, the degree of adiposity as assessed by the BMI was not associated with any measure of NREM sleep intensity and depth.

## Discussion

In this study, we performed a comprehensive and rigorous laboratory assessment of sleep quality in overweight and obese men and women recruited from the community. Our sample was not selected based on prior diagnosis or symptoms of OSA. We observed a high prevalence of OSA, which was present in two-thirds of the men and nearly half of the women. After adjustment for age, BMI and race/ethnicity, the odds ratio of having OSA was 3 times higher in men as compared to women. Our results also reveal that the sex differences in duration of SWS (43–46) and in intensity of NREM sleep by spectral analysis (8, 47) previously reported in lean populations are also present in overweight and obese adults with OSA. We found a differential impact of OSA on the intensity of NREM sleep in men vs. women. Indeed, after adjusting for potential confounders, there was a graded inverse relationship between the severity of OSA and SWA in men, but not in women. A previous cross-sectional study with a small sample size has shown that in men, the presence of OSA is associated with lower SWA (48), likely because of fragmentation and arousals induced by respiratory events. In women, the impact of OSA on SWA has not been previously characterized. A possible explanation for the observed sex differences in the impact of OSA on the intensity of NREM sleep could be that women tend to have REM-related OSA, i.e., the majority of the obstructive events during REM sleep, more often than men (49–51). However, this was not the case in our cohort and therefore would not explain our findings. Sex differences in NREM SWA have also been hypothesized to be due to difference in skull size and thickness, as well as skin thickness (52), but anatomic differences would also not explain the different impact of OSA on men's sleep compared to women's. Finally, differences in neuronal activity could exist between men and women. This has been suggested by a few studies assessing sex differences in the impact of aging on EEG activity, as well as findings in patients with affective disorders. In men, SWA as well as SWS decline after the third decade, while such a decline occurs later in women (8, 53, 54). Similarly, decreased levels of SWA have been reported in men with major depression, compared to controls, but this was not observed in women (55). The exact mechanisms underlying these differences have yet to be identified, but these and other studies suggest a greater “neural slow wave synchronization” ability in women than men (56). In our case, this could explain why women are able to reach SWS despite recurrent respiratory events as well as why OSA is less prevalent or less severe in premenopausal women despite higher levels of obesity when compared to men.

Another important novel finding is that, in our cohort of overweight and obese young to middle-aged men, higher testosterone levels were strongly associated with a lower intensity of NREM sleep. Similar results were obtained with free testosterone concentrations. This observation suggests that common genetic or non-genetic pathways may link the intensity of NREM sleep, a stable phenotype that is highly variable across individuals, and the set point of the hypothalamo-pituitary-gonadal axis. A potential consequence of this inverse association might be that high testosterone levels are associated with a lower arousal threshold and a greater vulnerability to respiratory instability. This interpretation is consistent with reports of increased severity of OSA following exogenous intramuscular testosterone administration (28–32), and further suggests that the exacerbation of OSA may occur through reductions in NREM SWA. It is noteworthy that the findings of our *cross-sectional* analysis are not in contradiction with the results of the few *intervention* studies that showed that CPAP treatment of OSA may increase testosterone levels (26, 57). Indeed, for any given male OSA patient in whom SWA has been fragmented by repeated complete or partial obstruction of the upper airway, the restoration of sleep continuity, particularly during NREM sleep, should be associated with enhanced nocturnal testosterone release.

In our cohort, despite the fact that they were older and heavier, men with OSA had slightly higher total testosterone levels compared to men without OSA. SHBG was slightly higher as well in the former. SHBG is known to increase with age and this could explain our findings (58). No differences in free testosterone were present between groups, and results of the multivariate analysis were unchanged when free testosterone was used as a covariate in our models. Of note, most of our participants had moderate OSA, while studies that reported decreased testosterone levels in men with OSA *vs*. controls included a majority of patients with severe OSA (25–27). Furthermore, participants enrolled in those studies were older than our volunteers [average age over 50 in all but two studies (27, 59)]. In these studies, OSA and control participants were matched for either BMI (25) or age (26, 27). Furthermore, in all studies that examined testosterone levels in men with OSA (26, 27, 60–64) except one (25), the average or median testosterone levels in the OSA group were in the normal range [>300 ng/dl (65)], suggesting that a majority of men with OSA do have normal testosterone levels.

We have to acknowledge the limitations of the present study. First, the cross-sectional nature of the study limits any assessment of causality. Second, the study protocol did not include a habituation night and we cannot exclude an impact of an unfamiliar sleeping environment on the sleep variables. However, this would have affected all groups equally. Third, our cohort is relatively small. Fourth, we could not systematically control for the menstrual phase in women (who were all premenopausal and off hormonal contraceptives). However, no impact of menstrual phase on NREM SWA levels has been detected in previous studies (66, 67). By not controlling for menstrual phase, we may also have underestimated the prevalence of OSA in women, since upper airway resistance is decreased in the luteal phase (68). Furthermore, we may have overestimated T levels in women as androgen concentrations have been shown to peak in the late follicular phase (69). Fifth, testosterone levels in women were measured by RIA, which is reported to be less accurate at low values. However, our assay has been validated against a liquid chromatography/tandem mass spectrometry method, with correlation coefficients of 0.97 for all samples (men and women), and 0.91 for samples obtained from women (36). Finally, our testosterone assay was performed on a single morning sample for each participant. However, our study was not aimed at diagnosing hypogonadism, which does requires measurement of testosterone levels on two separate occasions at least (65). Furthermore, only one morning sample was obtained in most other studies that examined the impact of OSA on androgen levels (26, 27, 60–64).

In summary, this cross-sectional analysis demonstrates a sex difference in the association between OSA severity and NREM SWA, as well as a robust negative association between total testosterone levels and intensity of NREM sleep in overweight and obese men. Further studies are needed to confirm and extend our results and elucidate the mechanisms linking circulating testosterone levels, SWA and OSA.

## Ethics statement

This study was carried out in accordance with the recommendations of the University of Chicago Institutional Review Board. The protocol was approved by the University of Chicago Institutional Review Board. All subjects gave written informed consent in accordance with the Declaration of Helsinki.

## Author contributions

EVC: designed the protocol; LLM, KAT, and RL: recruited subjects and collected data; LLM, EVC, and BM: analyzed the data; LLM: drafted the manuscript; EVC, DAE, and BM: reviewed and edited the manuscript. EVC and BM are both the senior authors for this submission.

### Conflict of interest statement

BM is supported by National Institutes of Health grant R01HL119161 and the Merck Investigator Studies Program. EV is the principal investigator of an investigator-initiated study sponsored by Philips/Respironics. The remaining authors declare that the research was conducted in the absence of any commercial or financial relationships that could be construed as a potential conflict of interest.
